# Drought and *Phytophthora* Are Associated With the Decline of Oak Species in Southern Italy

**DOI:** 10.3389/fpls.2018.01595

**Published:** 2018-11-05

**Authors:** Michele Colangelo, J. Julio Camarero, Marco Borghetti, Tiziana Gentilesca, Jonàs Oliva, Miguel-Angel Redondo, Francesco Ripullone

**Affiliations:** ^1^School of Agricultural, Forest, Food and Environmental Sciences, University of Basilicata, Potenza, Italy; ^2^Pyrenean Institute of Ecology (IPE-CSIC), Zaragoza, Spain; ^3^Department of Crop and Forest Sciences, Agrotecnio Center, University of Lleida, Lleida, Spain; ^4^Department of Forest Mycology and Plant Pathology, Swedish University of Agricultural Sciences, Uppsala, Sweden

**Keywords:** carbon isotopes, dendroecology, drought stress, forest dieback, *Phytophthora* species, *Quercus cerris*, *Quercus pubescens*, water-use efficiency

## Abstract

Forest decline induced by climate change is a global phenomenon that affects many tree species, mainly in drought-prone areas as the Mediterranean region. In southern Italy, several oak species have shown decline symptoms and elevated mortality since the 2000s due to drought stress. However, it remains to be answered whether decline occurred alone or whether a pathogen was also involved. To this aim, we compared two coexisting oak species in a forest located in southern Italy which are assumed to be less (*Quercus cerris*) and more tolerant to drought (*Quercus pubescens*). We sampled fifteen couples of neighboring declining (D) and non-declining (ND) trees of both species. Wood cores were taken from all trees to perform dendrochronological analyses to detect the decline onset and link it to potential climatic drivers. Carbon isotope ratios (d^13^C) were analyzed in wood of the two vigor classes to compare their water-use efficiency. *Phytophthora* presence was also assessed in soil samples from ten D-ND couples of trees per species. The oak species most affected by drought-induced decline in terms of leaf shedding and mortality was *Q. cerris*, i.e., the least tolerant to drought. In both species, the D trees showed a reduced growth rate compared with ND trees from 2000 onward when drought and warming intensified. *Q. pubescens* showed higher growth sensitivity to precipitation, temperature and drought than *Q. cerris*. This sensitivity to climate was magnified in D trees whose growth decreased in response to warm and dry conditions during the prior winter and the late summer. The *Q. pubescens* D trees were more efficient in their water use than ND trees before the growth divergence between D and ND trees amplified. In the studied area, *Phytophthora quercina* was isolated from 40% of the sampled trees, and tended to be more frequent amongst ND than amongst D trees. Our data suggests that droughts and warm summer conditions triggered oak decline. The high prevalence of *P. quercina* in the studied area warrants further study as a potential predisposing factor.

## Introduction

Forest decline is a complex phenomenon characterized by a loss in tree vigor and increased mortality, and it is usually triggered by extreme climate events (e.g., droughts) but also involves pathogens and pests (Manion, 1991; Sangüesa-Barreda et al., 2015).

In oaks, climate warming and altered precipitation regimes may negatively affect the carbon and water use of trees and indirectly benefit root pathogens (e.g., *Phytophthora* spp.) causing the dead of fine roots and making trees more susceptible to water shortage (Wargo, 1996; Führer, 1998; Thomas et al., 2002; Haavik et al., 2015). However, it remains to be answered to what degree severe climatic stressors (drought or heat waves) trigger forest decline making trees more vulnerable to biotic stressors (root pathogens).

Drought and high evapotranspiration rates linked to warmer conditions have been found to reduce radial growth, increase stress and trigger forest decline in many oak species worldwide (Tainter et al., 1990; Jenkins and Pallardy, 1995; Pedersen, 1998; Andersson et al., 2011; Corcuera et al., 2004a,b; Sohar et al., 2014). Comparisons of tree-ring width series through time has revealed that declining trees showed lower radial-growth rates than non-declining trees about 5–25 years prior to the decline onset or before tree death (Haavik et al., 2011; Levaniè et al., 2011; Helama et al., 2016; Cailleret et al., 2017; Gentilesca et al., 2017; Colangelo et al., 2017a, 2018). Declining trees are usually characterized by recent low growth rates but also by leaf shedding and architectural anomalies such as abundant epicormic and dead shoots (Drobyshev et al., 2007).

The most likely mechanism causing drought-induced decline in anisohydric species as many ring-porous oak species is hydraulic failure (McDowell et al., 2008). Such prolific use of water during drought leads to a reduction of stem water potential and an elevated loss in hydraulic conductivity of declining individuals showing intense crown desiccation and leaf shedding (Nardini et al., 2013). If unusually severe and sustained droughts modify C supply (photosynthesis, leaf area) and demand (growth, root production) in oaks, and alter aboveground and belowground C reserves and translocation, trees could become more susceptible to necrotrophic root-rot pathogens (Oliva et al., 2014) such as several *Phytophthora* species (Brasier, 1996; Jung et al., 1999, 2000). Drought and soil pathogens can synergistically amplify stress and cause the decline and death of oaks (Haavik et al., 2015).

In Europe, *Phytophthora quercina* has been commonly associated with oak root rot and decline predominantly in wet sites (Jung et al., 1999, 2000; Vettraino et al., 2002; Balci and Halmschlager, 2003; Jönsson, 2004). However, it remains unclear whether *Phytophthora* is the main trigger for oak decline episodes, because the relationships between environmental conditions, root damage caused by the pathogen and the loss in tree vigor are poorly understood (Jönsson et al., 2005; Jönsson, 2006). Although associations between oak crown transparency and the presence of *Phytophthora* species in the rhizosphere have been reported (Jung et al., 2000; Vettraino et al., 2002), information on the growth rates of coexisting non-declining and declining trees which show disease development is still missing (Jönsson et al., 2005).

Drought stress has been considered among the main triggering factors of oak decline, but it remains to be answered if both drought and pathogens synergistically contribute to the long-term loss in growth and tree vigor. Furthermore, to our best knowledge there are not experiments which investigated the effect of both stressors under controlled conditions on our studied species, except for other *Quercus* species (see for instance Sànchez et al., 2002 on *Q. ilex* where *P. cinnamomi* was investigated).

Here we use tree-ring width data to reconstruct growth and pinpoint the onset of decline in two affected oak species coexisting in the same forest located in southern Italy (*Quercus pubescens*, *Quercus cerris*). This comparison of the two species allows testing if the species more abundant in xeric sites (*Q. pubescens*) was less affected by drought-induced decline than the species from mesic sites which is assumed to be less tolerant to water shortage (*Q. cerris*). Both *Q. pubescens* and *Q. cerris* seem to be susceptible to *P. quercina*, because incidence of the pathogen has been previously reported in stands of these oak species (Vettraino et al., 2002; Balci and Halmschlager, 2003). Although differences in drought tolerance among oak species are widely reported in literature, there are only few comparative studies on Mediterranean oaks (e.g., Dreyer et al., 1992; Valentini et al., 1992; Tognetti et al., 1999). Based on the distribution of the main oak species in southern Italy, the following ranking in drought tolerance can be suggested: *Q. robur* (least tolerant) < *Q. cerris* < *Q. frainetto* < *Q. pubescens* < *Q. ilex* < *Q. suber* (most tolerant).

We hypothesize that the severe 2000s droughts triggered oak decline, and that *Phytophthora quercina* was involved in the decline syndrome either as predisposing or as a contributing factor. To test this idea, we reconstructed the tree-ring growth, isotopic signature of declining oaks and we assessed the association between the presence of symptoms of decline and *Phytophthora* infestation.

## Materials and Methods

### Study Area, Tree Species and Forest Decline

The study site (Gorgoglione forest, 40° 21′ 51″ N, 16° 10′ 34″ E, 800–850 m a.s.l.) is located in the mountainous Basilicata region, southern Italy. It has a mean slope of 25%. The study area is a mixed high forest with a mean density of 600 stems ha^-1^ dominated by *Quercus cerris* L. (71%) followed by *Quercus pubescens* L. (25%) and by other broadleaf species (4%). No recent disturbance has been reported for the study sites (e.g., insect outbreaks or fires) and no silvicultural treatment has been applied in the last five decades. In the past, these oak forests were traditionally managed as coppices for firewood and timber production in combination with livestock grazing. Soils are a mixture of sand, silt, and clay textures.

Climate in the study area is Mediterranean (cool dry-summer climate according to the Köppen classification), characterized by dry and warm summers (total precipitation from June to August is 93 mm) and wet and mild winters (total precipitation from December to February is 230 mm) with mean annual temperature of 11.6°C and annual precipitation of 722 mm (data from Gorgoglione station, 40° 24′ N, 16° 09′ E, 796 m). The warmest and coldest months are August (mean temperature of 20.5°C) and January (mean temperature of 3.7°C), respectively, whereas the driest and wettest months are July (22 mm) and November (96 mm). Drought occurs from June to August.

The two studied tree species showed recent drought-induced decline symptoms since the early 2000s (shoot dieback, leaf loss and withering, growth decline, and high mortality). Regarding the incidence of the decline syndrome according to local reports, annual oak mortality in the study area affected ca. 450 ha and shifted from 5 to 10% from 2002 to 2004. In the most affected stands, more than 50% of mature specimens showed decline symptoms and 15% were dead, while the remaining individuals (35%) were non-declining trees.

### Long-Term Climate and Drought Data

Due to the shortness and heterogeneity of local climate data we used gridded (0.25° resolution) climate data from the E-OBS dataset version 13.0 (Haylock et al., 2008) to quantify climate trends and climate-growth associations for the period 1950–2016. Climate was extracted from the 0.25° grid with coordinates 40.00–40.25° N, 16.00–16.25° E. To evaluate drought-growth associations we downloaded the Standardized Precipitation Evapotranspiration Index (SPEI) for the 0.5° grid where the study sites are located and considering 1–24 months long scales using the Global SPEI database webpage^1^. The SPEI is a multiscalar drought index, which considers the effects of temperature and evapotranspiration on drought severity and indicates wet (positive SPEI values) and dry (negative SPEI values) conditions (Vicente-Serrano et al., 2010).

### Field Sampling and Tree-Ring Data

In the field, first we randomly located seven circular plots (radius of 15 m) to estimate the density of trees with different vigor. Then, we sampled dominant trees of the two oak species. We selected pairs of neighboring (located at less than 20 m apart) declining or symptomatic (D trees) and non-declining or asymptomatic (ND trees). Declining oaks (hereafter D trees) were considered those with crown transparency higher than 50%, whereas non-declining oaks (hereafter ND trees) were considered those with transparency lower than 50%. Crown transparency was estimated by a visual assessment performed by two independent observations on the same tree using binoculars (Colangelo et al., 2017a). Using other crown-transparency thresholds (40 and 60%) the main results presented here did not change (cf. Colangelo et al., 2017a). Note, however, that recently dead trees could have been logged by people from nearby villages so our data may underestimate the actual density of D and recently dead trees.

The diameter at breast height (dbh), i.e., at 1.3 m, and height of each tree were measured using tapes and a laser rangefinder, respectively. To study radial growth we extracted one increment core per tree at breast height (1.3 m) using 5-mm Pressler increment borers. We avoided taking two cores per tree due to restrictions imposed by forest managers for preserving the study stands.

To quantify radial-growth changes in trees of contrasting vigor we used dendrochronology. In total we sampled 42 D and 32 ND *Quercus cerris* trees and 17 D and 21 ND *Quercus pubescens* trees. Within each ND-D couple, trees were located 10–15 m apart at maximum. Wood samples were air–dried and the surface of the cores was cut using a sledge core microtome (Gärtner and Nievergelt, 2010). Tree rings were visually cross-dated and measured with precision of 0.01 mm using a binocular microscope coupled to a computer with the LINTAB package (RINNTECH, Heidelberg, Germany). The COFECHA program (Holmes, 1983) was used to evaluate the visual cross-dating of tree-ring series. To estimate age at 1.3 m we counted the number of annual rings. In cores without pith, the missing distance was estimated by fitting a template of concentric circles with known radii to the curve of the innermost rings, which allowed the estimation of the missing radius length and transforming it into the number of missing rings (Camarero et al., 2015).

To quantify climate- and drought-growth relationships, first we removed the long–term trends of tree-ring width series by detrending them through the Friedman super smoother, which preserves high-frequency (annual) variability in the resulting ring-width indices. In addition, an autoregressive model was applied to each detrended series to remove most of the first-order autocorrelation related to the previous-year of growth. We obtained series at the tree level of dimensionless ring-width indices. Lastly, a biweight robust mean was used to obtain mean chronologies of ND and D trees at each site. Chronology development and standardization were carried out using the ARSTAN program (Cook and Krusic, 2005).

Climate-growth associations were calculated for the common and best-replicated period 1950–2016, considering monthly climatic variables (mean maximum and minimum temperatures, precipitation). The window of analyses included form the previous September to current September based on previous studies (Tessier et al., 1994). Drought-growth associations were calculated for the same time period using 1- to 24-month long SPEI values obtained from January to December.

Finally, we calculated dendrochronological statistics to compare the ring-width series of ND and D trees. Specifically, we calculated the first-order autocorrelation of ring-width data (AR1) and the mean sensitivity of ring-width indices (MS) which assesses the relative variability in width between consecutive rings (Briffa and Jones, 1990).

### Carbon Isotopes in Wood

We compared carbon isotope ratios (^13^C/^12^C, δ^13^C) in wood between non-declining (*n* = 5 trees) and declining (*n* = 5 trees) individuals of each species. We considered three different 5-year periods including different phased of the decline event (2002–2006, 2007–2011, and 2012–2016). The wood samples were dried in the oven at 70°C for 48 h and ground to a fine powder. Wood aliquots (0.001 g) were weighed on a microbalance (AX205 Mettler Toledo, OH, United States) into tin foil capsules and combusted to CO_2_ using a Flash EA-1112 elemental analyzer interfaced with a Finnigan MAT Delta C isotope ratio mass spectrometer (Thermo Fisher Scientific Inc., MA, United States). Isotope analyses were conducted at the Isotope Lab of the “Istituto di Geoscienze e Georisorse” (CNR; Udine, Italy). Stable isotope ratios were expressed as per mil deviations using the δ notation relative to Vienna Pee Dee Belemnite (VPDB). The standard deviation for repeated analyses was better than 0.1‰

To account for the δ^13^C depletion of atmospheric CO_2_ (δ^13^*Ca*) due to the combustion of fossil fuels, we calculated C isotope discrimination in wood (Δ^13^C) from δ^13^Ca and plant δ^13^*Cp* following Farquhar et al. (1989):

(1) ΔC13 =(δ13Ca−δ13Cp)/(1+δ13Cp/1000)

δ^13^*Ca* was obtained from Graven et al. (2017).

### Soil Characteristics

Soils were sampled at ten points separated by 10 m and located within the sampled stands. Soils were taken at a depth of 15–30 cm. Soils were characterized them by measuring the following variables: texture, C and N concentrations, pH and electrical conductivity. Soil texture was determined with laser diffraction method in Coulter Mastersizer (2000) and clay content was corrected (Taubner et al., 2009). Soil C and N concentrations and the C/N ratio were determined with an elemental analyzer (Elementar Vario MAX N/CM, Hanau, Germany).

### Detection of Phytophthora Presence

We focused on the detection of *Phytophthora* since this pathogen has been often associated with decline and mortality of oak stands across Europe (Jung et al., 1999, 2000). To evaluate whether dieback was associated with soilborne *Phytophthora* infection we sampled the rhizosphere of twenty trees (five defoliated and five healthy trees ×2 tree species). Approximately 400 gr of shallow soil were collected after removing the organic layer from three points situated below the crown of each oak taken at a mean 25 cm depth in the A horizon. Samples were pooled for each tree. Soil baiting was carried out following Jung’s (2009) using fresh leaves of Valencian oak (*Quercus faginea*) as baits. Baits were inspected regularly during 10 days. Necrotic spots were plated on CMA-PARPBH medium (Jeffers and Martin, 1986) and then incubated at 20°C in darkness. Within 2–4 days, *Phytophthora*-like colonies were transferred onto V8 agar media (Erwin and Ribeiro, 1996), and stored at 20°C. DNA from 14 *Phytophthora*-like isolates was extracted following the NaOH extraction procedure described by Wang et al. (1993). The ITS region was amplified using the ITS6f and ITS4 primers (White et al., 1990; Cooke and Duncan, 1997), following the PCR settings specified in Samils et al. (2011). PCR product was sequenced by Macrogen (South Korea), and a BLAST search was later performed on the Phytophthora ID curated database^2^, establishing a minimum identity threshold for species identification of 98%.

### Statistical Analyses

Tree characteristics of the two tree vigor classes were compared between species and between vigor classes using *t* or Mann-Whitney *U* tests in the case of variables that followed or did not follow normal distributions, respectively. To assess trends in monthly climate data (mean maximum and minimum temperatures, precipitation) we used the non-parametric tau statistic (τ) considering climate data for the 1950–2016 period. We used the Wilcoxon rank-sum test to check if mean tree-ring width differed between ND and D trees because this non-parametric statistic is robust against deviations from normal distributions and the presence of temporal autocorrelation (Gibbons and Chakraborti, 2011).

## Results

### Climate Trends and Drought Variability

**FIGURE 1:**
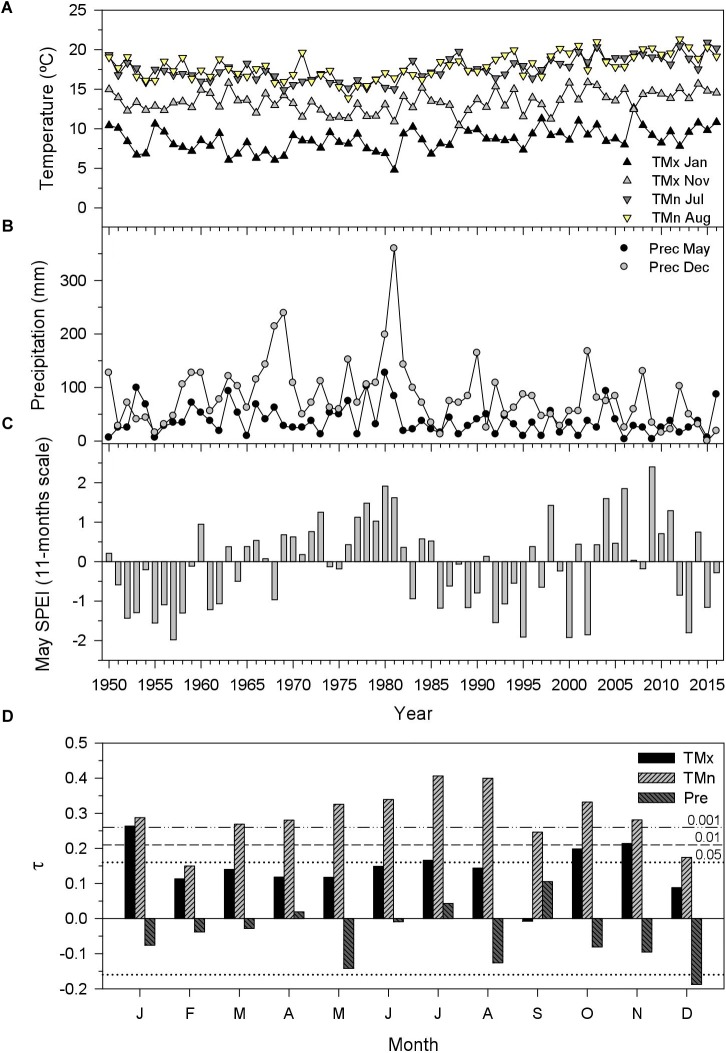
Climate trends and drought patterns in the study area. The selected climate variables correspond to monthly mean maximum (TMx) and minimum (TMn) temperatures **(A)** and precipitation [**(B)** Prec] showing the most pronounced trends for the 1950–2016 period as assessed through the tau (τ) statistic **(D)**. The drought severity was assessed using the May Standardized Precipitation Evapotranspiration Index (SPEI) for 11-months long scales **(C)**, which showed high associations to tree growth (see Figure 4).

Significant (*P* < 0.05) and positive trends in mean maximum temperatures of January (τ = 0.26), July (τ = 0.17), October (τ = 0.20), and November (τ = 0.21) were found for the period 1950–2016 (Figure 1). All months excepting February showed significant and positive trends in mean minimum temperatures, being particularly strong in July–August (τ = 0.40). Only December showed a significant negative trend in precipitation (τ = -0.18), followed by May (τ = -0.14). Lastly, the May SPEI calculated at 11-month long scales showed dry conditions (SPEI < -1.75) in 1957, 1995, 2000, 2002, and 2013, whereas wet conditions (SPEI > 1.75) occurred in 1980, 2006, and 2009.

### Density and Size of Declining and Non-declining Trees

The densities of D trees were 262 trees ha^-1^ (64% of trees) and 43 trees ha^-1^ (30% of trees) in *Q. cerris* and *Q. pubescens*, respectively (Table 1). ND trees had thicker stems (greater dbh) and were taller than D trees in both species, but age did not differ between the two vigor classes (Table 1).

**Table 1:** Main structural characteristics of the non-declining (ND) and declining (D) oak trees sampled in Gorgoglione forest.

Tree species	Vigor class	Density (trees ha^-1^)	Diameter at 1.3 m (cm)	Height (m)	Age at 1.3 m (years)
*Quercus pubescens*	ND	100 ± 11b	26.5 ± 1.3b	11.3 ± 0.3b	115 ± 4
	D	43 ± 6a	22.5 ± 0.8a	8.6 ± 0.3a	111 ± 3
*Quercus cerris*	ND	148 ± 13a	33.0 ± 0.9b	15.0 ± 0.6b	102 ± 4
	D	262 ± 16b	28.3 ± 0.7a	12.2 ± 0.4a	107 ± 2


*Values are means ± SE. Different letters indicate significant differences at the 0.05 level based on *t*-tests.*

### Growth Patterns and Comparisons of the Two Vigor Classes

**FIGURE 2:**
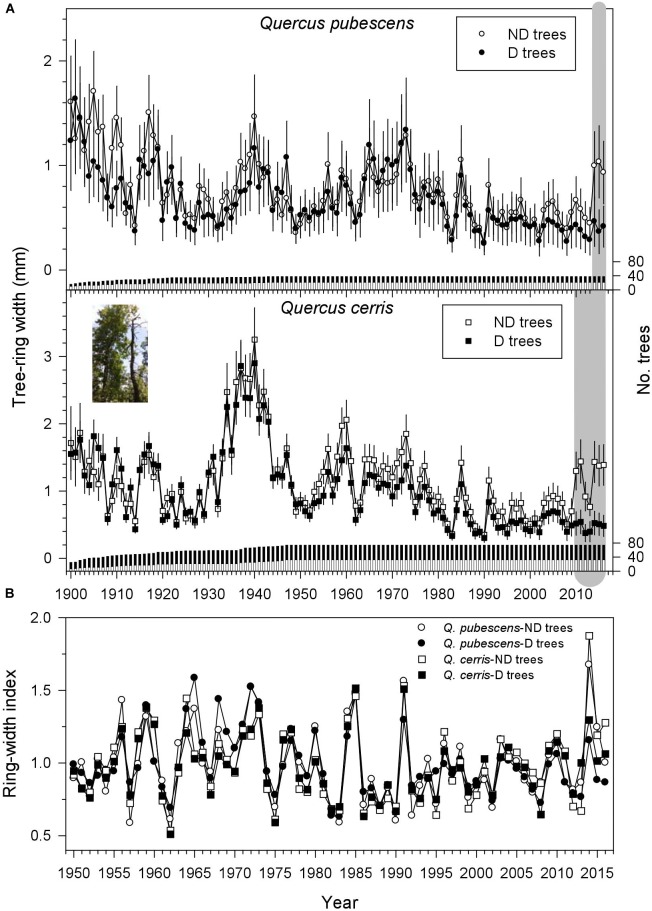
Radial-growth patterns from 1900 to 2016 of non-declining (ND) and declining (D) trees in **(A)** *Q. pubescens* and *Q. cerris* and **(B)** residual ring-width chronologies for the best-replicated period (1950–2016). The right *y* axes show the sample depth. In the plot **(A)** the gray areas indicate periods when tree-ring width of ND trees was significantly (*P* < 0.05) higher than in D trees according to Wilcoxon rank-sum tests. Values are mean tree-ring widths ± SE. The inset shows one of the sampled couples of ND and D *Q. cerris* trees.

Radial growth increased in the 1901s, from the 1930s to 1940s, in the late 1950s and 1960s, in the and early 1970s, and in the 2000s and 2010s; but it decreased in the 1920s, early 1950s and early 1960s, 1980s, and 1990s (Figure 2A). Tree-ring width was higher in ND than in D trees for the periods 2014–2016 and 2010–2016 in *Q. pubescens* and *Q. cerris*, respectively (Figure 2A). Considering the common period (1950–2016) ND trees had wider rings than D trees (*Q. pubescens*, 0.94 vs. 0.73 mm; *Q. cerris*, 1.22 vs. 1.07 mm), whilst the rest of dendrochronological statistics did not differ between the two vigor classes (Table 2). It is also remarkable the higher correlations of the individual series with the mean series of each vigor class observed in *Q. cerris* as compared with *Q. pubescens*, indicating a higher coherence and a higher responsiveness to climate of the former species as compared with the latter.

**Table 2:** Tree-ring width statistics of the non-declining (ND) and declining (D) oak trees sampled in Gorgoglione forest and calculated for the common and best-replicated period 1950–2016.

Tree species	Vigor class	Time span	Tree-ring width (mm)	AR1	MS	Correlation with mean series
*Quercus pubescens*	ND	1881–2016	0.94 ± 0.10b	0.66 ± 0.03	0.32 ± 0.01	0.45 ± 0.03
	D	1883–2016	0.73 ± 0.04a	0.63 ± 0.04	0.33 ± 0.01	0.50 ± 0.04
*Quercus cerris*	ND	1890–2016	1.22 ± 0.04b	0.69 ± 0.02	0.34 ± 0.01	0.67 ± 0.02
	D	1879–2016	1.07 ± 0.04a	0.72 ± 0.01	0.34 ± 0.01	0.66 ± 0.02


*Values are means ± SE. Different letters indicate significant differences at the 0.05 level based on *t*-tests. Variables’ abbreviations: AR1, first-order autocorrelation of ring-width data; MS, mean sensitivity of ring-width indices.*

During the common period 1950–2016, sharp growth reductions in both species and vigor classes (e.g., 1957, 1962, 1983, 1986, 2002, and 2012–2013) usually corresponded to dry conditions, whereas growth increases corresponded to wet conditions (1960, 1972–1973, 1977, 1980, 1985, 1998, 2004, and 2009; compare Figures 1C, 2B). An abrupt increase of the ring-width index was observed in ND trees of both species in 2014. The four ring-width chronologies were highly and significantly correlated during 1950–2016 suggesting a high coherence and similar responses to climate (Figure 2B), but the highest and lowest correlations were observed for *Q. cerris* ND and D trees (*r* = 0.90, *P* < 0.001) and for *Q. cerris* ND and *Q. pubescens* D trees (*r* = 0.68, *P* < 0.001), respectively.

### Growth Responses to Climate and Drought

**FIGURE 3:**
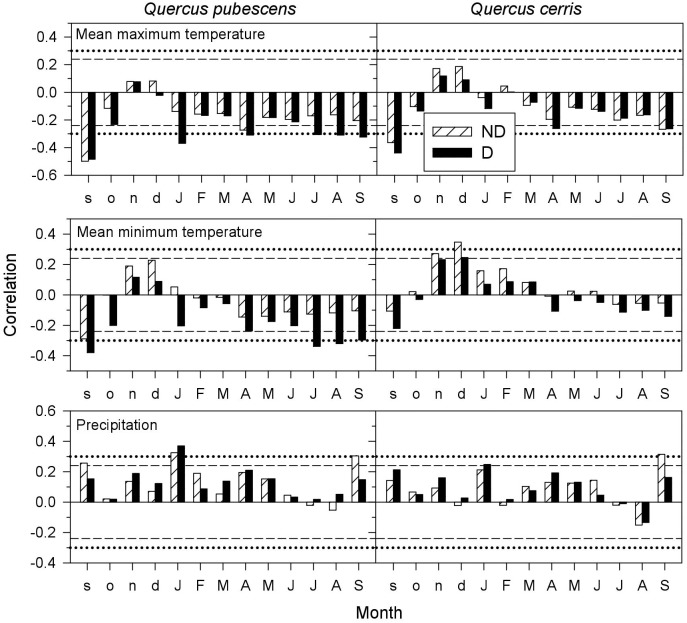
Climate-growth relationships (Pearson correlations) in declining (D, filled bars) and non-declining (ND, hatched bars) trees of *Quercus pubescens* and *Quercus cerris*. The dashed and dotted horizontal lines indicate the 0.05 and 0.01 significance levels.

Both *Q. pubescens* and *Q. cerris* growth responded negatively to warm previous September conditions, regardless the tree vigor (Figure 3). However, the growth of *Q. pubescens* D trees was sensitive to high maximum and minimum temperatures in January, April and from current July to September. In *Q. cerris*, D trees were more sensitive to warm April conditions, whilst warm conditions in current September were negatively associated to growth of both vigor classes. In *Q. cerris*, high minimum temperatures in the prior late autumn and early winter (November and December) enhanced growth of ND trees. Wet January conditions benefitted growth of both species, particularly of D trees, whereas wet current September conditions (and also in the prior September for *Q. pubescens*) improved growth of ND trees.

**FIGURE 4:**
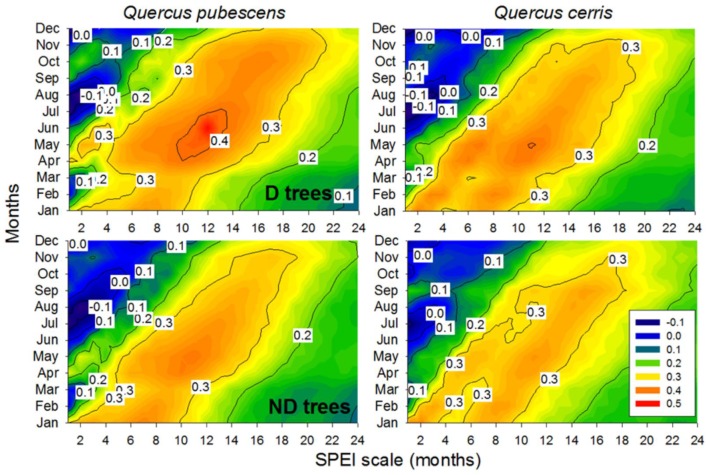
Drought-growth relationships (Pearson correlations) in declining (D) and non-declining (ND) trees of *Quercus pubescens* and *Quercus cerris*. The color scale shows the correlations between ring-width indices and the Standardized Precipitation Evapotranspiration Index (SPEI) calculated at 1–24 months long scales (*x* axes) from January to December (*y* axes). Significance levels are as in Figure 2.

In all species and vigor classes, the maximum positive correlations between ring-width indices and SPEI (*r* = 0.37–0.42) were found for May and considering 11-months long scales, i.e., from the previous July up to May (Figure 4) The highest correlations with May SPEI calculated at 11-months scales were found for *Q. pubescens* D trees (*r* = 0.42). In *Q. cerris* ND trees, very high correlations (*r* = 0.38) were also observed with January SPEI and considering a 2-month scale.

### Soil Features and Phytophthora Presence

Soils were of silt loam texture with mean percentages of 55, 32, and 13% for silt, sand and clay. The mean (±SE) soil C and N concentrations were 3.21 ± 0.24 and 0.17 ± 0.01%, respectively (mean C/N ratio is 20.5 ± 4.0). The soil pH was 7.5 ± 0.1 and the soil electrical conductivity was 184.0 ± 8.7 ms cm^-1^ indicating slight salinity.

We isolated *Phytophthora quercina* in 40% of the sampled trees. Phytophthora tended (*p* = 0.068, Chi-square test without Yates correction) to be more frequent on ND than on D trees (same proportion was observed for *Q. pubescens* and *Q. cerris*). The low number of infected trees per defoliation class did not allow the comparison of tree ring width between D and ND trees. Nevertheless, comparison of tree-ring width data between *Phytophthora*-infected and -uninfected trees (regardless of the defoliation class) within each tree species for the periods 2014–2016 and 2010–2016 did not reveal significant differences (*Q. pubescens*, Mann-Whitney *U* = 81–149, *P* = 0.41–0.62; *Q. cerris*, Mann-Whitney *U* = 271–319, *P* = 0.43–0.69; see Supporting Information, Supplementary Figure S1).

### Wood C Isotopes

Regardless the vigor class, *Q. pubescens* showed higher d^13^C values (mean ± SE = -27.33 ± 0.22‰) than *Q. cerris* (-27.45 ± 0.19‰), but differences between the two species were not significant (*t* = 0.42, *P* = 0.67). Considering the two vigor classes, D trees (-26.85 ± 0.27‰) showed significantly higher d^13^C values than ND trees (-27.79 ± 0.29‰) in *Q. pubescens* (*t* = 2.35, *P* = 0.026), but differences were not significant in the case of *Q. cerris* (D trees, -27.39 ± 0.30‰; ND trees, -27.53 ± 0.25‰; *t* = 0.36, *P* = 0.72; Table 3). Considering different 5-year periods, d^13^C and Δ^13^C were higher in D than in ND trees of *Q. pubescens* for the period 2007–2011.

**Table 3:** Wood C isotope radios (d^13^C) and discrimination (Δ^13^C) in non-declining (ND) and declining (D) *Q. cerris* and *Q. pubescens* individuals considering three 5-year periods.

Tree species	Period	d^13^C (‰)		D^13^C (‰)	
			
		ND trees	D trees	ND trees	D trees
*Quercus cerris*	2002–2006	-26.92 ± 0.45	-26.76 ± 0.39	19.17 ± 0.41	19.33 ± 0.47
	2007–2011	-27.77 ± 0.41	-27.57 ± 0.48	19.90 ± 0.50	20.11 ± 0.43
	2012–2016	-27.99 ± 0.32	-27.83 ± 0.64	20.01 ± 0.67	20.32 ± 0.30
*Quercus pubescens*	2002–2006	-27.14 ± 0.51	-26.08 ± 0.36	18.45 ± 0.37	19.56 ± 0.53
	2007–2011	-27.85 ± 0.42a	-26.73 ± 0.28b	19.02 ± 0.30a	20.19 ± 0.45b
	2012–2016	-28.38 ± 0.52	-27.94 ± 0.37	19.99 ± 0.33	20.54 ± 0.54


*Different letters indicate significant (*P* < 0.05) differences between ND and D trees within each period based on *t*-tests.*

## Discussion

The study area has experienced a rise in minimum temperatures since 1950 and this warming trend coincided with the occurrence of severe droughts in the early 2000s and early 2010s. As expected, these warm and dry conditions probably triggered the oak decline phenomena affecting more *Q. cerris* (less tolerant to drought) than *Q. pubescens* (more tolerant to drought). Similarly, Gentilesca et al. (2017), in a recent review, reported drought as the main driver triggering oak decline within the Mediterranean area, although other causes (i.e., increasing temperature, low temperature, tree nutritional status, overaging, excessive stand density or pathogens attack) could have also exacerbated decline.

In this study, the oak decline was characterized by conspicuous leaf shedding and high mortality rates which were preceded by recent divergences in radial growth between ND and D trees (2010–2016 in *Q. cerris*, 2014–2016 in *Q. pubescens*).

Our findings agree with previous studies in the region which found that ND trees were larger (thicker diameter, longer stems) than D trees (Colangelo et al., 2017a,b). However, they do not support global reviews suggesting that the tallest trees are more vulnerable to drought-induced hydraulic failure (Bennett et al., 2015). This discrepancy may be explained by several factors making smaller, but dominant, trees more vulnerable to drought damage. First, it is possible that oaks forming short stems develop more shallow roots or do not have access to deep soil-water pools, but this aspect remains understudied. Second, tree height is related to the size of the stem sapwood water reserves and may determine hydraulic capacitance and vulnerability to drought (Zhang et al., 2009).

Considering the period 1950–2016, ND trees presented slightly higher growth rates than D trees in both oak species (29 and 14% higher in *Q. pubescens* and *Q. cerris*, respectively). The divergence in growth between both vigor classes was recent, thus indicating that the growth reduction started about 3–7 years in *Q. pubescens* and *Q. cerris*, respectively, before the decline intensified and trees started to die. This pattern indicates that slow-growing trees were prone to show decline and does not concur with other studies showing that trees growing vigorously produce vessels of wider lumen and are more prone to xylem embolism and decline (Levaniè et al., 2011; Voltas et al., 2013). In *Quercus rubra* currently D trees were growing at slower rates than ND trees but D trees grew more in the past than ND trees suggesting that rapid growth early in development led to later decline and mortality (Haavik et al., 2011). However, we did not observe this pattern characterized by D oaks showing rapid growth early in life. The growth increase in the 1930s, which was more evident in *Q. cerris* than in *Q. pubescens*, suggests past management in the study forest, probably logging of trees for timber or firewood. This past legacy effects could interact with recent climate warming if formerly logged trees were growing better and were also less vulnerable to drought damage (Camarero et al., 2011), but this potential interaction merits further research.

As previously observed, we also found that radial growth of Mediterranean deciduous oaks is enhanced by mild and wet spring conditions and that the decline was triggered by severe droughts and warm conditions during the growing season (Tessier et al., 1994; Corona et al., 1995; Amorini et al., 1996; Haavik et al., 2011; Alla and Camarero, 2012). Climate-growth associations were usually stronger in non-declining than in declining trees, particularly in the sensitive *Q. pubescens*, which agrees with Romagnoli et al. (2018). In oaks growth responses to drought are often lagged 1 year and they are characterized by leaf shedding, shoot dieback and the lack of latewood production leading to altered patterns of hydraulic conductivity (Corcuera et al., 2004a,b; Di Filippo et al., 2010). We also observed those patterns but working with mature and dominant individuals in a high forest, whereas other studies on the decline of Mediterranean oak species using dendroecology were developed on coppice stands (e.g., Amorini et al., 1996; Di Filippo et al., 2010). Spring drought was the main triggering factor of dieback in these studies where declining trees often showed lower growth rates than non-declining trees prior (about 20 years before in *Q. cerris*) to the onset of the decline episode (Amorini et al., 1996). In recently dead *Q. robur* trees, growth of dying trees was also lower than in surviving trees 1–25 years prior to death (Andersson et al., 2011). By contrast, here the ND-D divergence in growth was recent and lasted from 3 (*Q. pubescens*) to 7 (*Q. cerris*) years. These different periods indicate distinct periods of stress and complex interactions between climate stress (drought) and perhaps opportunistic pathogens causing dieback and tree death (Marcais and Bréda, 2006).

The higher responsiveness to year-to-year climate variability of *Q. pubescens* compared to *Q. cerris* corresponds to the highest vulnerability to drought damage of *Q. cerris*. These results agree with previous dendroecological research (Garfi, 2000; Cherubini et al., 2003). It is noticeable that *Q. pubescens* growth mainly responded to January precipitation whereas *Q cerris* growth responded more to warm November-December mean minimum temperatures suggesting a higher sensitivity of *Q. cerris* to warm winter conditions. This could explain the vulnerability to drought of *Q. cerris* since we detected the most marked decrease of precipitation and increase in maximum temperatures during winter. Interestingly, the growth responsiveness to climate was more marked in D than in ND trees of *Q. pubescens* trees and the growth loss in response to warm night conditions from July to August observed in D confirms their highest sensitivity to summer drought as confirmed the SPEI-growth analyses.

*Q. pubescens* shows higher and more variable radial-growth rates than *Q. cerris* and also a better post-drought resilience or recovery capacity (Tognetti et al., 2007). According to these authors both oak species are able tolerate some drought stress but *Q. pubescens* better tolerates water shortage than *Q. cerris* by uptaking deep soil water and using it more efficiently. Overall, this suggests that *Q. pubescens* can better tolerate drought stress than *Q. cerris* in terms of wood production and associated processes (carbon uptake and storage, hydraulic conductivity, fine root production, etc.).

We isolated *Phytophthora quercina* from the rhizosphere of 40% of the trees that we sampled. Whether the observed prevalence may pose a threat for these stands is unknown, but the fact that our isolation frequency of *P. quercina* was four times higher than the one reported in the north of Italy (Vettraino et al., 2002), may suggest that further monitoring could be carried out. The fact that soil sampling was carried out in winter, when the pathogen is less active, may have underestimated its true incidence, and could indicate that *P. quercina* is even more widespread than reported. *P. quercina* is a primary and aggressive pathogen, which causes root necrosis and dieback of fine roots leading to tree decline and death (Jung et al., 1999). In this area, *P. quercina* was observed more frequently in healthy than in declining trees, indicating that the pathogen did not seem to play a crucial role in the observed decline as a contributing factor. However, the tendency to isolate the pathogen from healthy trees suggests that *P. quercina* could instead have played a role in the onset of decline, as a predisposing factor (*sensu* Manion), although the lower number of samples collected in this survey requires caution about the possible role played by the pathogen. In other ecosystems in which *Phytophthora* spp. also act as primary pathogens, a similar pattern has been observed. For instance, *P. alni* was more frequently isolated from cankers in relatively symptomless alder trees rather than from highly symptomatic trees (Redondo et al., 2015). Further studies comparing the frequency of decline in areas with and without *P. quercina* should be carried out in order to confirm the role of *P. quercina* as predisposing factor. Considering not only the presence, but other more quantitative measures of inoculum could also help elucidating the role of this pathogen in the decline of this area. In parallel, managers could improve forest resilience against *Phytophthora* attacks by improving soil drainage and restricting further spread or by selecting genotypes resistant to this soil-borne pathogen (Sena et al., 2018).

The carbon isotope discrimination (Δ^13^C) was used as surrogate of intrinsic water-use efficiency (*WUEi*), i.e., the ratio between the photosynthesis (*A*) and the stomatal conductance (*g*) rates (cf. Ripullone et al., 2004). The D trees of *Q. pubescens* present a higher *WUEi* than conspecific ND trees for the period 2007–2011 when the growth divergence between D and ND trees increased. This could be explained because those D trees presented a persistent growth reduction in response to warm and dry conditions during the growing season but at the same time reduced *g* by forcing stomatal closure, and probably showed a reduction in hydraulic conductivity as is common in anisohydric oak species (Granda et al., 2017). It is unclear why the difference in *WUEi* between D and ND *Q. pubescens* trees diminished in the most recent period but it could be a common response to severe dry conditions regardless the vigor class. By contrast, D trees showed lower *WUEi* than ND trees after the dieback onset in *Quercus frainetto*, which was associated to higher *g* and water loss through transpiration (Colangelo et al., 2017a). In *Q. robur*, dying trees had also lower *WUEi* than surviving trees (Levaniè et al., 2011), which was interpreted as lower *A* or to higher *g* (Farquhar et al., 1989). The difference in *WUEi* between ND and D trees was found in *Q. pubescens* but not in *Q. cerris* which can be attributed to their different ecophysiological behaviors. When these species coexist, *Q. pubescens* shows a more conservative water-use strategy than *Q. cerris*, since *Q. pubescens* presents higher *WUEi*, and lower photosynthesis rates, stomatal conductance rates and negative midday water potential than *Q. cerris* (Tognetti et al., 2007). These complex responses related to *WUEi* indicate the need to increase the samples collection and more studies considering different decline episodes, using several functioning (growth, wood anatomy) and ecophysiological proxies (C and O isotope discrimination) and involving tree species with diverse susceptibility to drought and pathogen damage (Gessler et al., 2018).

## Conclusion

Drought and warmer minimum temperatures triggered oak decline in the 2000s and 2010s. Such drier conditions affected more negatively *Q. cerris* than *Q. pubescens*. The more tolerant *Q. pubescens* was also the most responsive to climate and drought in terms of wood production, particularly declining trees. The sensitive *Q. pubescens* declining trees showed higher water-use efficiency than non-declining trees of the same species before the growth divergence between these two vigor classes started in 2014. *Phytophthora quercina* was isolated from both oak species, and its incidence tended to be higher in non-declining than in declining trees, suggesting a possible role as predisposing factor. However, considering the reduced number of individual surveyed in this field experiment further studies are needed to support this hypothesis. These findings illustrate complex responses to drought and soil-borne pathogens of coexisting oak species suggesting different tolerances to the combined effects of climate and biotic stressors under the forecasted warmer and drier conditions.

## Author Contributions

MC, JC, MB, TG, and FR conceived the idea and contributed to writing the manuscript. MC, TG, and FR carried out the field work. The dataset was analyzed by JC, MC, and JO. *Phytophthora* analyses were carried out by JO and M-AR. All authors contributed to the interpretation of the results and the revision, discussion, and approval of the final draft.

## Conflict of Interest Statement

The authors declare that the research was conducted in the absence of any commercial or financial relationships that could be construed as a potential conflict of interest.

## Footnotes

1 http://sac.csic.es/spei/database.html

2 http://phytophthora-id.org
