# Supplementary material for: Drought and Phytophthora Are Associated With the Decline of Oak Species in Southern Italy
Source: Front Plant Sci. 2018 Nov 5;9:1595. doi: 10.3389/fpls.2018.01595 (PMC6230577; doi:10.3389/fpls.2018.01595)
Supplement: Supplementary file 1 [file Table_1.DOCX]

Supplementary Material

**Drought and *Phytophthora* contribute to the decline of oak species in southern Italy**

Michele Colangelo^1,2^*, J. Julio Camarero^2^, Marco Borghetti^1^, Tiziana Gentilesca^1^, Jonàs Oliva^3,4^, Miguel-Angel Redondo^4^, Francesco Ripullone^1^

*, corresponding author (e-mail: [michelecolangelo3@gmail.com](mailto:michelecolangelo3@gmail.com))

## Supplementary Figures

**Figure S1.** A comparison of tree-ring width series of non-declining (ND trees, grey lines), declining (D trees, black lines) and trees where *Phytophthora quercina* was isolated (red lines) in the two study oak species.

**
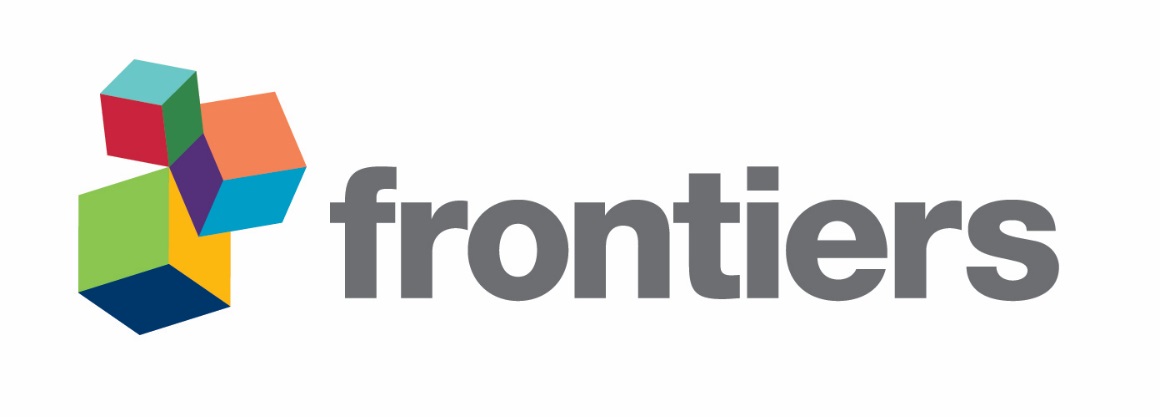
**
